# Response of Coastal Fishes to the Gulf of Mexico Oil Disaster

**DOI:** 10.1371/journal.pone.0021609

**Published:** 2011-07-06

**Authors:** F. Joel Fodrie, Kenneth L. Heck

**Affiliations:** 1 Institute of Marine Sciences and Department of Marine Sciences, University of North Carolina at Chapel Hill, Morehead City, North Carolina, United States of America; 2 Dauphin Island Sea Lab and Department of Marine Sciences, University of South Alabama, Dauphin Island, Alabama, United States of America; National Oceanic and Atmospheric Administration/National Marine Fisheries Service/Southwest Fisheries Science Center, United States of America

## Abstract

The ecosystem-level impacts of the Deepwater Horizon disaster have been largely unpredictable due to the unique setting and magnitude of this spill. We used a five-year (2006–2010) data set within the oil-affected region to explore acute consequences for early-stage survival of fish species inhabiting seagrass nursery habitat. Although many of these species spawned during spring-summer, and produced larvae vulnerable to oil-polluted water, overall and species-by-species catch rates were high in 2010 after the spill (1,989±220 fishes km-towed^−1^ [μ ± 1SE]) relative to the previous four years (1,080±43 fishes km-towed^−1^). Also, several exploited species were characterized by notably higher juvenile catch rates during 2010 following large-scale fisheries closures in the northern Gulf, although overall statistical results for the effects of fishery closures on assemblage-wide CPUE data were ambiguous. We conclude that immediate, catastrophic losses of 2010 cohorts were largely avoided, and that no shifts in species composition occurred following the spill. The potential long-term impacts facing fishes as a result of chronic exposure and delayed, indirect effects now require attention.

## Introduction

Prevailing models of ecological impacts resulting from oil pollution are being revised after the April 2010 release of ∼4.4 million barrels [1] of oil into the northern Gulf of Mexico (GOM). In part, this is a legacy of the Exxon Valdez accident as a watershed environmental catastrophe, and the extensive research on acute and chronic impacts of the resulting inshore oil pollution [2]. Unlike the 0.25–0.5 million barrels released by the Valdez [2], however, the Deepwater Horizon (DH) disaster hemorrhaged oil into the open ocean at 1500 m depth over a protracted 84-day period [1]. As a critical step toward new model development applicable for detecting impacts of the DH spill, rigorous observational data at organismal through community levels are needed to guide ecosystem-based toxicology.

We have already learned that a significant fraction of the oil released into the GOM from the Macondo well did not rise to the surface, and this has implications for the ecosystem-level responses we should anticipate. Rather, oil was emulsified at the well head due to turbulent mixing, reduced buoyancy at depth, and addition of Corexit 9500 dispersant. Subsequently, mid-water hydrocarbon plumes [3] have been observed with stimulation of petroleum-degrading bacteria [4]. With this now understood, we revisit some early concerns regarding impacts for nearshore fisheries [5].

During the DH spill, near-surface waters lacked any reliable refuge from oil pollution, as slicks/sheens occurred at the immediate surface and oil was emulsified throughout the water column. For many fishes, including commercially valuable snappers (Lutjanidae) and groupers (Serranidae), spawning occurs during the spring or summer (table S1), and eggs, larvae and post-larvae would have relied upon near-surface waters overlaying the continental shelf during the DH spill [6]–[7]. Furthermore, eggs/larvae and oil can be transported by the same hydrodynamic and atmospheric processes, enhancing the probability of oil encounters for many species. Because the population ecology of marine species with bipartite life histories is disproportionately affected by the health and survival of early life stages [8], understanding how eggs, larvae and newly-settled juveniles coped with the DH spill is essential for quantifying ecosystem responses.

We hypothesized that the strength of juvenile cohorts spawned on the northern GOM continental shelf during May–September 2010 in the northern GOM would be negatively affected by egg/larval-oil interactions. Oiled seawater contains toxic compounds such as polycyclic aromatic hydrocarbons (PAHs) which, even after weathering, can result in genetic damage, physical deformities and altered developmental timing for fish eggs/larvae [9]–[10]. These effects may be induced at very low (∼1 ppb PAHs) levels of exposure when persistent over days to weeks [11]–[12] - timescales relevant for larval development and descriptive of the DH spill. Additionally, emulsified oil droplets could mechanically damage the feeding and breathing apparatus of relatively fragile larvae and further decrease individual fitness. Unfortunately, observing egg/larval mortality, growth or migration *in situ* is an enduring challenge for biological oceanographers, as eggs/larvae are simply too dilute and experience relatively high instantaneous mortality, even in undisturbed systems [13].

**Figure 1:**
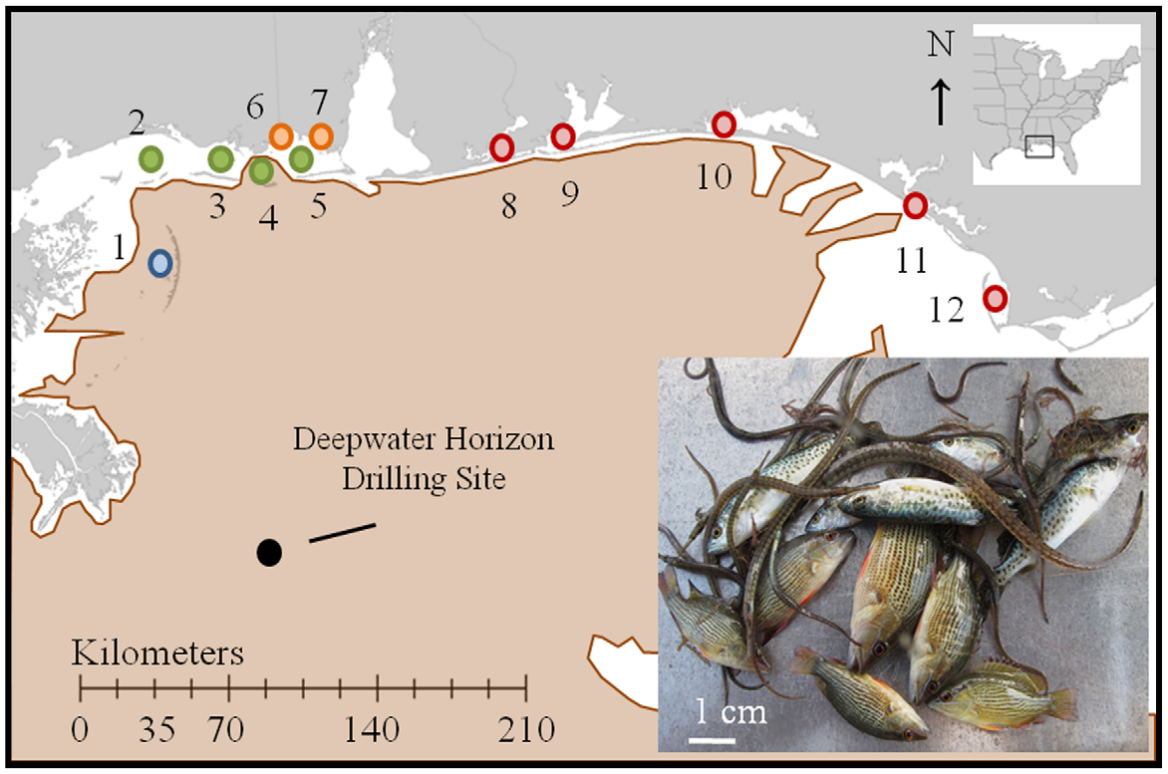
Sampling region and study sites. Map of juvenile fish sampling stations, divided among four survey areas: Chandeleur Islands (blue circles), Gulf Islands (green circles), Grand Bay (orange circles) and Florida Bays (red circles). 1. Chandeleur Is., LA; 2. Ship Is., MS; 3. Horn Is., MS; 4. Petit Bois Is., MS; 5. Dauphin Is., AL; 6. Grand Bature Shoal, AL; 7. Point Aux Pines, AL, 8. Big Lagoon, FL; 9. Pensacola Bay, FL; 10. Choctawhatchee Bay, FL; 11. St. Andrew Sound, FL; 12. St. Joseph Bay, FL. The spread of surface oil during the 84-day spill is also shown (brown shading). Image at lower right shows juvenile gray snapper (*L. griseus*), spotted seatrout (*C. nebulosus*) and pipefish (*Syngnathus* spp.).

In the absence of direct observations on eggs and larvae, juvenile abundance data provide valuable indices of the acute, population-level responses of young fishes to the spill. Although indirect evidence [14], early juvenile abundances are the integrated products of early life-history processes such as fertilization, larval growth/mortality, and settlement [6]–[8]. Therefore, effects of oil pollution on early life stages should be detectable in time series data as shifts in the abundance of recently settled juvenile fishes. We tested these predictions using 2006–2010 survey data collected from the Chandeleur Islands, LA, to Saint Joseph Bay, FL (Fig. 1), representing most of the nearshore region directly impacted by oil. In contrast to the difficulties of surveying marine larvae, quantitative measures of juvenile abundances are tractable due to the tendency of settled fish to aggregate in specialized nursery habitats [15]. In the northern GOM, many fish species, such as those in the drum (Sciaenidae), snapper and grouper families have juveniles that are routinely collected from shallow-water seagrass meadows they use as primary nurseries [16].

Our dataset consisted of 853 individual trawl samples taken between July 15 and October 31 of 2006–2010 within seagrass meadows of the northern GOM (tables S2, S3). We collected 167,740 individual fishes representing 86 taxa, and examined catch-per-unit-effort (CPUE) data for all species pooled together, as well as separately for each of the 20 most abundant species. We also tested for post-spill community-level shifts in seagrass-associated fish assemblages using multivariate analyses [17]. We recognized that not all species were at equal risk for oil exposure due to variation in spawning timing and larval distributions (tables S1, S4). Furthermore, some species may have experienced release from fishing pressure due to large-scale fishery closures [18] during the spill (table S5), perhaps enhancing their larval production during the summer spawning season. Therefore, we also considered how these factors affected species-specific CPUEs during 2010. In all analyses, comparisons among years were considered as a proxy for the effects of oil disturbance (2006–2009 as undisturbed, 2010 as disturbed).

## Results

**Figure 2:**
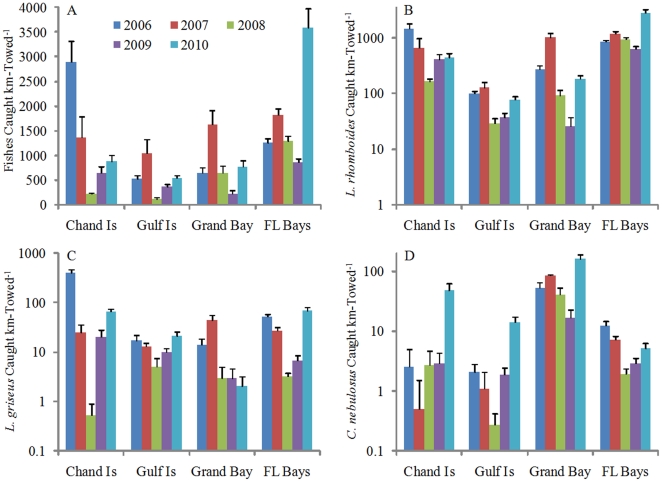
Catch rates of juvenile fishes, 2006–2010. Catch rates among years and sampling areas (Chandeleur Islands, Gulf Islands, Grand Bay and Florida Bays) for: (A) all fishes pooled; (B) pinfish (*L. rhomboides*), (C) gray snapper (*L. griseus*), and (D) spotted seatrout (*C. nebulosus*). CPUE data in panels B–D are presented on a log scale, and all data are shown as means of trawl samples (μ + 1SE).

Within the oil-affected GOM, a five-year survey of seagrass-associated fish communities did not indicate reductions in juvenile abundances following the spill. Rather, of the twenty most commonly collected fish species, twelve were characterized by statistically higher catch rates in 2010 relative to 2006–20009 (α = 0.05; Table 1). Among the remaining eight taxa, pre- and post-spill catch rates were statistically indistinguishable. Across our entire study region, CPUE increased from 1,080±43 fishes km-towed^−1^ (μ ± 1SE) during 2006–2009 to 1,989±220 fishes km-towed^−1^ in 2010. When resolved among four geographical areas (Chandeleur Islands, Gulf Islands, Grand Bay, Florida Bays; Fig. 1), overall catch rates of juvenile fishes, as well as CPUE of the most abundant species, pinfish (*Lagodon rhomboides*), were consistently higher during 2010 than in 2008 or 2009, and in some areas were higher in 2010 than all previous years (Fig. 2A–B; fig. S1, S2, S3; table S6).

**Table 1:** Relative frequencies and CPUE data for abundant fishes collected during sampling in seagrass meadows of the northern GOM.

Scientific name	Cumlative % Freq	2006–09 CPUE	2010 CPUE	*P* (df = 851)	Trend	Risk of oil encounters	Potential release from fishing pressure
*Lagodon rhomboides*	60.22	644.86	1379.32	<0.001	**↑**	Moderate-Low	No
*Eucinostomus* spp.	72.67	119.94	60.21	0.086	**NC**	Low	Potential Bycatch
*Bairdiella chrysoura*	82.66	123.10	163.77	0.117	**NC**	Moderate-Low	No
*Orthopristis chrysoptera*	89.90	80.31	118.73	0.007	**↑**	Moderate	Potential Bycatch
*Lutjanus griseus*	91.64	23.63	43.02	0.003	**↑**	High	Yes
*Stephanolepis hispidus*	93.29	11.95	70.61	<0.001	**↑**	High	No
*Lutjanus synagris*	94.68	14.79	19.18	0.171	**NC**	High	Yes
*Cynoscion nebulosus*	95.74	13.41	36.51	<0.001	**↑**	Low	Yes
*Syngnathus* spp.	96.63	11.57	20.05	0.057	**NC**	Moderate-Low	No
*Chilomycterus schoepfi*	97.46	7.37	18.56	<0.001	**↑**	Unknown	No
*Leiostomus xanthurus*	97.78	4.63	2.56	0.533	**NC**	Moderate-Low	Potential Bycatch
*Opsanus beta*	98.04	2.73	6.63	<0.001	**↑**	Low	No
*Arius felis*	98.29	2.62	10.14	0.021	**↑**	Low	Potential Bycatch
*Nicholsina usta*	98.52	2.11	6.86	0.003	**↑**	Unknown	No
*Sphoeroides* spp.	98.70	2.26	2.24	0.974	**NC**	Low	No
Blenniidae	98.86	2.06	5.27	0.002	**↑**	Low	No
*Mycteroperca microlepis*	99.01	1.96	1.75	0.773	**NC**	High	Yes
*Paralichthys* spp.	99.16	1.97	2.90	0.133	**NC**	Moderate	Yes/ Potential Bycatch
*Archosargus probatocephalus*	99.31	1.58	5.95	<0.001	**↑**	Unknown	Yes
*Lactophrys quadricornis*	99.43	1.47	3.22	0.036	**↑**	Unknown	No

Trend symbols indicate no change (NC) or statistically significant increase (↑) in catch rates during 2010 relative to 2006–2009. Risk of oil encounters determined by spawning season and across-shelf larval distribution for each species. Potential release from fishing pressure guided by state and federal management plans, as well as shrimp-trawl bycatch data (SI).

**Figure 3:**
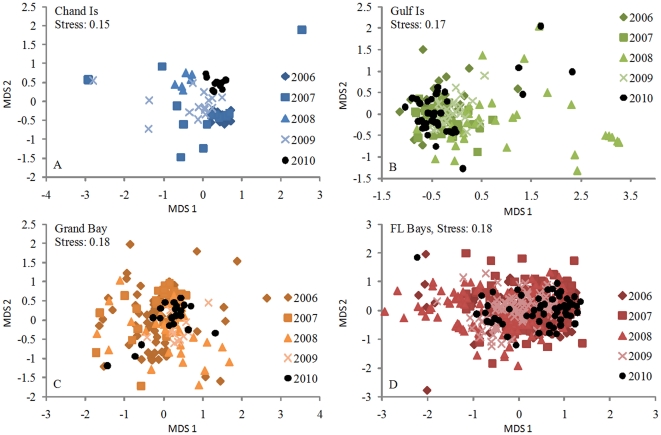
Community composition of seagrass-associated fish communities, 2006–2010. Multi-dimensional scaling plots for seagrass-associated fish communities prior to (2006–2009; colored symbols) and following (2010; black circles) the DH spill. Data for (A) Chandeleur Islands, (B) Gulf Islands, (C) Grand Bay and (D) Florida Bays are presented separately. Each datum represents a single trawl sample.

The species composition of juvenile fish assemblages was unaltered in each sampling area during the months following the DH disaster (Fig. 3). Prior to the spill, similarities among individual trawl samples (SIMPER) ranged from 50.3% at the Chandeleur Islands to 52.9% within Florida Bays (table S7). By comparison, similarity percentages between pre- (2006–2009) and post-spill (2010) samples were correspondingly high, ranging from 43.4% within Grand Bay to 50.8% at the Chandeleur Islands. Furthermore, pinfish, silver perch (*Bairdiella chyrsoura*), mojarras (*Eucinostomus* spp.), pigfish (*Orthopristis chrysoptera*) and spotted seatrout (*Cynoscion nebulosus*) drove similarity patterns both before and after the spill (table S7). Species richness (S, up 15%, *p*<0.001), diversity (ES_(20)_, up 11%, *p* = 0.006; H′, up 18%, *p*<0.001) and evenness (J′, up 11%, *p* = 0.003) among trawl samples were all slightly elevated during 2010 relative to 2006–2009 averages (table S8; fig. S4), indicating that high CPUEs in 2010 were broad based.

**Figure 4:**
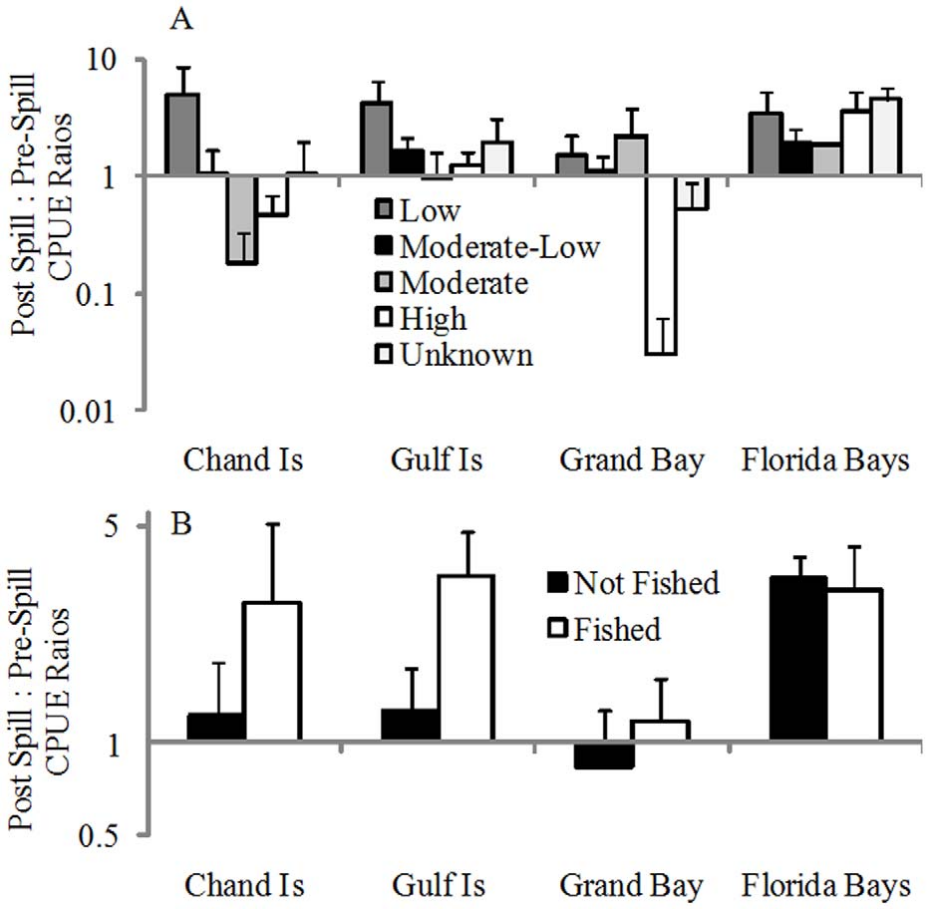
Larval risk and fishery closure impacts. Effects of (A) egg/larval vulnerability and (B) harvest pressure on the responses of fishes to the DH spill. Response of individual species calculated as the ratio of 2010 versus 2006–2009 CPUE data. Data are presented on a log scale as group means (μ + 1SE), with ratios >1 indicating that 2010 catch rates were elevated relative to 2006–2010 data.

When averaged across species, there was little statistical evidence that either exposure risk or release from fishing pressure significantly affected CPUEs during 2010. When comparing 2010 CPUE data against pre-spill catch rates, we did observe that fishes characterized by moderate (spring spawning, nearshore larvae) or high risk (spring-summer spawning, larvae distributed across the continental shelf) exhibited decreases in CPUE following the spill at the Chandeleur Islands and Grand Bay (Fig. 4A). However, no statistically significant differences were found as a function of egg/larval risk (F_4,848_ = 1.410, *p* = 0.242) or sampling areas (F_3,849_ = 0.999, *p* = 0.440; table S9). Similarly, release from fishing pressure on spawning fishes could be implicated, although not proven, as a determinant of post-spill CPUEs. Along the Chandeleur and Gulf Islands, increases in catch rates during 2010 relative to 2006–2009 were 800% and 950% higher, respectively (Fig. 4B), for species identified in state and federal management plans than for species not harvested by fishermen (table S5). No similar patterns were recorded within Grand Bay or Florida Bays, however, and effects of fishing pressure (F_1,851_ = 1.510, *p* = 0.223) and area (F_3,849_ = 1.397, *p* = 0.225) on CPUE responses were not significant.

## Discussion

Collectively, no significant, acute impacts on the strength of juvenile cohorts within seagrass habitats were detected following the DH disaster. This was true for all species examined, bolstering our confidence in the conclusion that ecosystem-level injuries were not severe for this community of fishes. Unfortunately, our assessment cannot be compared to the most analogous spill, the IXTOC 1 blowout [5], due to a paucity of formal scientific investigation following that accident (The 1979 IXTOC I blowout at 3600 m depth, 80-km north of the Yucatán Peninsula, was a ∼3.5-million-barrel spill.). The most parsimonious explanation for our data is that these fishes were resilient to the spill, possibly due to the retention of a large proportion of spilled oil at depth. As such, these data add to a developing literature [3]–[4] in which the acute impacts of the spill may be concentrated in the deep ocean rather than shallow-water, coastal ecosystems that were the focus of early concern [5]. For instance, gray snapper (*Lutjanus griseus*) larvae were abundant in surface waters (0–25-m deep) over the continental shelf from July through September [19], and were among the most likely individuals to have contacted oil-polluted water. Still, catch rates of gray snapper juveniles following the spill were high relative to the four previous years (up 82%, Fig. 2C; area * pre/post spill context interaction *p*<0.001, table S6).

When averaged across species - and in some cases across species with vastly different life histories - there were no statistically significant differences in the response of fished or unfished species to the spill (or their responses to subsequent management actions; i.e., fishery closures). Still, there were notable patterns suggesting that certain species may have been released from harvest pressure during 2010, subsequently enhancing spawning activity and post-spill cohort sizes despite any potentially negative oil impacts. For example, spotted seatrout spawn during summer [20], but many mature individuals are typically removed by recreational fishers before reproducing. Following the fishery closures in 2010, we recorded order-of-magnitude higher juvenile abundances of spotted seatrout at the Chandeleur and Gulf Islands, as well as elevated catch rates throughout our survey region (Fig. 2D; area, pre/post spill context and 2-way interaction *p*<0.001, table S6).

Consistent with the patterns observed in the species-by-species catch data and analyses of ‘risk” or ‘fishing” effects, there were no significant post-spill shifts in community composition and structure, nor were there changes in any of several biodiversity measures. While natural recruitment variability can make it difficult to detect population-level impacts for any one species following large-scale disturbance [14], our whole-community analyses and results are likely robust against these concerns.

Several other factors could have contributed to the high catch rates of seagrass-associated fishes in 2010 despite large-scale oil pollution. For instance, fishes may be uniquely buffered against oil pollution due to their mobility or foraging ecology [21]–[22]. Also, the major predators of fish eggs/larvae (e.g., gelatinous zooplankton) may have been impacted by the spill, thereby reducing natural mortality rates for coastal fishes [23]. Regardless of the mechanism(s) involved, thus far the potential for 2010 cohorts to support regional fisheries over the next several years has persisted despite the spill. This information is critical for projecting the mode and tempo of ecological and economic recovery in the oil-affected GOM, as well as guiding future conservation/restoration activities to mitigate oil-spill injuries.

While these data provide reason for early optimism, attention should now turn to possible chronic effects of oil exposure on fishes as well as delayed indirect effects cascading through the post-spill GOM. Fish may suffer growth, survival or reproductive penalties years after exposure to oil [24], or experience altered migratory behaviors [25]. Oil sequestered in sediments may also affect species laying benthic eggs for several years [26]. More broadly, ecosystems experiencing large-scale disturbance can carry or build instabilities over protracted periods that can eventually result in delayed collapses of fisheries stocks [27].

Improved threat assessment for energy exploration [28] and process-oriented studies of ecosystem responses will be long-term initiatives resulting from the DH spill. In the short term, however, observational data collected over relevant spatial and temporal scales are invaluable for guiding and evaluating targeted studies of oil toxicology [29]. For fish species experiencing multiple stressors such as habitat degradation [30] harvest pressure [31], climate change [16], and now oil pollution, rigorous baseline survey data and new syntheses are needed to enact effective ecosystem-based management.

## Materials and Methods

### Sampling

We analyzed changes in northern Gulf of Mexico (GOM) seagrass-associated fish communities during the last 5 years by comparing survey data obtained either prior to (2006–2009) or immediately following the Deepwater Horizon disaster (2010). The survey region extended approximately 340 km along the coastline, including a significant portion of the inshore area most affected by oil (Fig. 1.). Each year, we made repeated sampling trips to 12 sites, extending from the Chandeleur Islands, LA, to St. Joseph Bay, FL (29.68–30.72°N, 85.30–89.10°W). Sampling occurred within mixed seagrass meadows that serve as primary nursery habitat for many juvenile fishes that have recently settled from the water column following a 5–45 day larval period [6], [16]. Our samples were collected from seagrass mosaics that included turtle grass (*Thalassia testudinum*), shoal grass (*Halodule wrightii*), widgeon grass (*Ruppia maritima*), and manatee grass (*Syringodium filiforme*), along with scattered unvegetated patches (table S3).

During each year, trawls were conducted from July 15 through October 31 in order to record the abundances and composition of fishes during the period when seagrass meadows are utilized as primary nurseries by recently settled juveniles (refer to table S1 for reproductive seasons of common fishes). Fishes were collected using a 5-m otter trawl (2.0-cm body mesh; 0.6-cm bag mesh; 0.3×0.7-m doors) with conventional 4-seam balloon design including float and lead lines but without tickler chains. Trawls consisted of 3.9±0.1 (μ ± 1SE) minute tows behind small (<7 m) research vessels traveling at 3.3+0.1 kilometers hour^−1^. Overall, 853 samples were taken (table S2), and the trawl covered a linear distance of 184.7 kilometers during our sampling. These trawls occurred in depths of 0.5–2.5-m, and were conducted during daylight hours. During our surveys, species were enumerated in the field unless species-level identifications were not easily made. Unidentified specimens were transported to the lab where meristics were used by at least two different technicians to identify each individual. In cases in which species could not be identified, specimens were classified to the lowest taxonomic level possible. Typically, fishes were 20–100 mm long (standard length), indicative of recently-spawned juveniles. However, we did not record individual sizes for all species, and, for pipefishes (*Syngnathus spp.*) and hard-headed catfish (*Arius felis*), we did observe that a small proportion of our catch included reproductive adults. For four species: gray snapper (50.5±0.8 mm [μ ± SE]), lane snapper (*Lutjanus synagris*; 55.7±0.7 mm), spotted seatrout (60.8±1.1 mm) and gag grouper (*Mycteroperca microlepis*; 157.5±3.2 mm); we did record the sizes of all individuals throughout our surveys. Based on our own otolith analyses (Fodrie unpublished) and published reports of first-year growth among these four species (age-1 sizes: gray snapper ∼109 mm; lane snapper ∼140 mm; spotted seatrout ∼127 mm; gag grouper >198 mm), we calculated that >96% of individuals were captured in the same year as they were spawned (including 2010).

Once enumerated, fishes were entered in to an Excel database, and abundance data were converted into catch-per-unit-effort (CPUE) data based on the linear distance over with each trawl occurred. All statistical analyses were applied to these CPUE data. Our complete CPUE dataset is included as a separate appendix in our supporting information (dataset S1). This study was carried out in strict accordance with the recommendations in the Guide for the Care and Use of Laboratory Animals of the National Institutes of Health. Our sampling protocol was approved by the Committee on the Ethics of Animal Experiments of the University of North Carolina at Chapel Hill (Permit Number: 10-114.0).

### Statistical analyses

We investigated differences in the catch rates of seagrass-associated fishes (all species pooled as well as the 20 most abundant species individually) by unpaired t-tests comparing pre- (2006–2010) and post-spill (2010) data (Table 1), as well as 2-way ANOVAs in which sampling area (Chandeleur Islands, Gulf Islands, Grand Bay, Florida Bays) and context (pre- versus post-spill) were fixed factors (table S6). Regions were defined by basic geomorphology and location, local water clarity, local salinity, and local seagrass composition [32]. Because variances were stable among groups, no data transformations were required prior to analyses.

We analyzed similarities and differences in fish communities among years (2006–2009 versus 2010) within each sampling area (each area considered separately) using non-metric multidimensional scaling (MDS), based on Bray-Curtis similarity indices among all individual trawl samples (4^th^ root-transformed data). Pairwise comparisons between trawl samples across years were conducted with analysis of similarity (ANOSIM) and similarity (or dissimilarity) percentages (SIMPER) using PRIMER 5.2.2 software (PRIMER-E Ltd; [33]).

We also examined patterns of species diversity among regions and years by computing the following measures for each trawl sample: ***S***, number of species collected; ***ES_(20)_***, species richness rarefied to 20 individuals; ***H′***, Shannon-Weiner diversity index (*log_e_*); and ***J′***, Pielou's evenness measure (PRIMER 5.2.2 software). We investigated differences in community diversity via 2-way ANOVAs in which sampling area (Chandeleur Islands, Gulf Islands, Grand Bay, Florida Bays) and context (pre- versus post-spill) were fixed factors. Because variances were stable among groups, no data transformations were required prior to analyses.

These approaches are proscribed in earlier syntheses for detecting environmental impacts [17]. Critiques of employing parametric testing to detect ecosystem injury exist due to interannual variability and reduced statistical power [14], although those concerns have focused on analyses involving single species.

We determined the relative probability for oil-larvae encounters (‘risk’) for the twenty most commonly captured fishes, and used these data to explore how individual species responded differently to large-scale oil pollution in the northern GOM. Information on the seasonal timing of spawning and distribution of larvae from shore to the outer margin of the continental shelf was collected from the literature (tables S1 and S4), and used to define 4 levels of risk (in addition to an ‘unknown’ [n = 4] category containing species for which no data were available). ‘Low’ risk species (n = 6) included those in which larvae remained inside estuaries, either in the plankton or as benthic egg masses, regardless of spawning season. ‘Moderate-Low’ risk species (n = 4) were defined by having either 1) larvae distributed in estuaries as well as across the continental shelf, or 2) larvae distributed across the continental shelf, but not likely during the spill period (i.e., April–September). Only two ‘Moderate’ risk species were identified: pigfish (*Orthopristis chrysoptera*) spawn throughout summer, and have larvae distributed within nearshore waters; while flounder (*Paralichthys* spp.) have larvae distributed across the continental shelf, with a protracted spawning that extends into April or May (i.e., some overlap with the oil spill). ‘High’ risk species (n = 4) spawn offshore and have larvae distributed across the continental shelf. Furthermore, spawning data indicates that these species would have produced larvae sometime during the DH spill (April–Sept in our classification scheme). Based on these risk guilds, we examined the response of fishes to the spill by calculating the ratio of 2010 CPUE data (averaged) to 2006–2009 CPUE data (averaged) for each species. Following these calculations, ratios >1 indicate that average 2010 catch rates were higher than during the previous 4 years, while ratios <1 indicate that average 2010 catch rates were lower than during the previous 4 years. Using each species as a replicate measure, we used ‘risk’ and region (Chandeleur Islands, Gulf Islands, Grand Bay, Florida Bays) as fixed factors in a 2-way ANOVA that compared 2010 CPUE: 2006–2009 CPUE trends. Because variances were stable among groups, no data transformations were required prior to analyses.

Similarly, we determined whether species were likely to have experienced significant release from harvest pressure following large-scale closures in the northern GOM, and examined how this may have affected CPUE data in 2010. For each of the twenty most commonly caught fish, we designated species as ‘fished’ if they were included in any state or federal management plan as of Dec 31, 2010 (table S5), or identified as <1% (by biomass) of bycatch in shrimp trawl fisheries within the northern GOM (table S5). As before, we examined the response of fishes to the spill by calculating the ratio of 2010 CPUE data (averaged) to 2006–2009 CPUE data (averaged) for each species. Using each species as a replicate measure, we used ‘fishing pressure’ (with fished species including species that are targeted or captured as incidental bycatch at significant levels) and region (Chandeleur Islands, Gulf Islands, Grand Bay, Florida Bays) as fixed factors in a 2-way ANOVA that compared 2010 CPUE: 2006–2009 CPUE trends. Because variances were stable among groups, no data transformations were required prior to analyses.

All univariate tests were conducted using StatView 5.0.1 software (SAS Institute Inc.). Because each statistical analysis applied to separate and easily distinguishable hypotheses, we made no corrections to experiment-wise alpha for any of the univariate (total fishes CPUE, individual fishes CPUE, risk guilds, harvest guilds, diversity) or multivariate (ANOSIM) tests we conducted [34].

## Supporting Information

Figure S1 Catch rates of all fishes, pooled together, among sampling areas prior to (2006–2009) and following (2010) the Deepwater Horizon disaster.
(DOCX)

Figure S2 Catch rates of individual species, among sampling areas prior to (2006–2009) and following (2010) the Deepwater Horizon disaster. Data are presented for the 20 most abundant species.
(DOCX)

Figure S3 Catch rates among sampling areas and years for the 20 most abundant species collected during trawl surveys.
(DOCX)

Figure S4 Diversity measures for seagrass-associated fish communities within sampling areas affected by the Deepwater Horizon disaster.
(DOCX)

Table S1 Summary table for CPUE data (fish kilometer-towed^−1^) of fishes prior to (2006–2009) and following (2010) the DH disaster.
(DOCX)

Table S2 Distribution of trawl samples among sampling areas (Chandeleur Islands, Gulf Islands, Grand Bay, Florida Bays) and years (2006–2010).
(DOCX)

Table S3 Quantitative description of seagrass habitats sampled throughout the northern Gulf of Mexico during 2006–2010.
(DOCX)

Table S4 Information used to determine the likelihood of larvae contacting oiled water during the summer of 2010.
(DOCX)

Table S5 Summary table for the management status of the 20 most abundant fishes collected during our survey program.
(DOCX)

Table S6 Summary table of the effects of sampling area and year (context: pre- versus post-spill) on the catch rates of the 20 most abundant fishes collected during surveys in northern Gulf of Mexico seagrass meadows.
(DOCX)

Table S7 Comparisons of community structure between catch data prior to (2006–2009) or immediately following (2010) the Deepwater Horizon disaster (ANOSIM and SIMPER).
(DOCX)

Table S8 Summary table of the effects of sampling area and year (context: pre- versus post-spill) on the diversity (S, ES_(20)_, H′, and J′) of trawl samples collected within northern Gulf of Mexico seagrass meadows.
(DOCX)

Table S9 Summary table of the effects of sampling area, larval risk and harvest pressure on the change in catch rates of individual species for pre- (2006–2009) and post-spill (2010) data.
(DOCX)

Dataset S1 Complete CPUE data obtained for 2006–2009 trawl surveys within seagrass meadows of the northern Gulf of Mexico.
(XLSX)
