# Supplementary material for: Response of Coastal Fishes to the Gulf of Mexico Oil Disaster
Source: PLoS One. 2011 Jul 6;6(7):e21609. doi: 10.1371/journal.pone.0021609 (PMC3130780; doi:10.1371/journal.pone.0021609)
Supplement: Figure S1 — Catch rates of all fishes, pooled together, among sampling areas prior to (2006–2009) and following (2010) the Deepwater Horizon disaster. (DOCX) [file pone.0021609.s001.docx]

Fig S1. Catch rates (μ + 1SE) of all fishes, pooled together, among sampling areas prior to (2006-2009) and following (2010) the Deepwater Horizon disaster.
