# Supplementary material for: Response of Coastal Fishes to the Gulf of Mexico Oil Disaster
Source: PLoS One. 2011 Jul 6;6(7):e21609. doi: 10.1371/journal.pone.0021609 (PMC3130780; doi:10.1371/journal.pone.0021609)
Supplement: Figure S2 — Catch rates of individual species, among sampling areas prior to (2006–2009) and following (2010) the Deepwater Horizon disaster. Data are presented for the 20 most abundant species. (DOCX) [file pone.0021609.s002.docx]

Fig S2. Catch rates (μ + 1SE) of individual species, among sampling areas prior to (2006-2009) and following (2010) the Deepwater Horizon disaster. Data are presented for the 20 most abundant species.

Fig. S2 – continued.
