# Supplementary material for: Response of Coastal Fishes to the Gulf of Mexico Oil Disaster
Source: PLoS One. 2011 Jul 6;6(7):e21609. doi: 10.1371/journal.pone.0021609 (PMC3130780; doi:10.1371/journal.pone.0021609)
Supplement: Figure S3 — Catch rates among sampling areas and years for the 20 most abundant species collected during trawl surveys. (DOCX) [file pone.0021609.s003.docx]

Fig S3. Catch rates (μ + 1SE) among sampling areas and years for the 20 most abundant species collected during trawl surveys. Refer to Table S6 for statistical comparisons of catch data.

Fig S3 – continued.
