# Supplementary material for: Response of Coastal Fishes to the Gulf of Mexico Oil Disaster
Source: PLoS One. 2011 Jul 6;6(7):e21609. doi: 10.1371/journal.pone.0021609 (PMC3130780; doi:10.1371/journal.pone.0021609)
Supplement: Figure S4 — Diversity measures for seagrass-associated fish communities within sampling areas affected by the Deepwater Horizon disaster. (DOCX) [file pone.0021609.s004.docx]

Fig. S4. Diversity measures for seagrass-associated fish communities within sampling areas affected by the Deepwater Horizon disaster (μ + 1SE). S = species collected in each trawl; ES_(20)_ = species richness rarefied to 20 individuals; H’ = Shannon-Weiner diversity index (*log_e_*); J’ = Pielou’s evenness measure. Refer to Tables S8for statistical comparisons among catch data.
