# Supplementary material for: Response of Coastal Fishes to the Gulf of Mexico Oil Disaster
Source: PLoS One. 2011 Jul 6;6(7):e21609. doi: 10.1371/journal.pone.0021609 (PMC3130780; doi:10.1371/journal.pone.0021609)
Supplement: Table S1 — Summary table for CPUE data (fish kilometer-towed−1) of fishes prior to (2006–2009) and following (2010) the DH disaster. (DOCX) [file pone.0021609.s005.docx]

Table S1. Summary table for CPUE data (fish kilometer-towed^-1^, μ + 1SE [underneath, parentheses]) of fishes prior to (2006-2009) and following (2010) the DH disaster. Also shown are t-test results comparing pre- and post-spill catch rates, as well as descriptions of the species-by-species potential for oil-larvae interactions and release from fishing pressure during the summer of 2010.
