# Supplementary material for: Response of Coastal Fishes to the Gulf of Mexico Oil Disaster
Source: PLoS One. 2011 Jul 6;6(7):e21609. doi: 10.1371/journal.pone.0021609 (PMC3130780; doi:10.1371/journal.pone.0021609)
Supplement: Table S2 — Distribution of trawl samples among sampling areas (Chandeleur Islands, Gulf Islands, Grand Bay, Florida Bays) and years (2006–2010). (DOCX) [file pone.0021609.s006.docx]

Table S2. Distribution of trawl samples among sampling areas (Chandeleur Islands, Gulf Islands, Grand Bay, Florida Bays) and years (2006-2010).
