# Supplementary material for: Response of Coastal Fishes to the Gulf of Mexico Oil Disaster
Source: PLoS One. 2011 Jul 6;6(7):e21609. doi: 10.1371/journal.pone.0021609 (PMC3130780; doi:10.1371/journal.pone.0021609)
Supplement: Table S3 — Quantitative description of seagrass habitats sampled throughout the northern Gulf of Mexico during 2006–2010. (DOCX) [file pone.0021609.s007.docx]

Table S3. Description of seagrass habitats (μ + 1SE [underneath, parentheses]) sampled throughout the northern Gulf of Mexico during 2006-2010. Sites are described in the SOM text and listed in Fig. 1.
