# Supplementary material for: Response of Coastal Fishes to the Gulf of Mexico Oil Disaster
Source: PLoS One. 2011 Jul 6;6(7):e21609. doi: 10.1371/journal.pone.0021609 (PMC3130780; doi:10.1371/journal.pone.0021609)
Supplement: Table S4 — Information used to determine the likelihood of larvae contacting oiled water during the summer of 2010. (DOCX) [file pone.0021609.s008.docx]

Table S4. Information used to determine the likelihood of larvae contacting oiled water during the summer of 2010 (see Table 1 and table S3 for summary status of larval risk for each species). Full citations are included in the reference list below [1-23].

1. Hildebrand SF, Cable LE (1930) Development and Life History of Fourteen Teleostean Fishes at Beaufort, N.C. Fish. Bull. **40**, 384 .

2. Hildebrand, Cable LE (1938)Further Notes on the Development and Life History of Some Teleosts at Beaufort, N.C. Fish. Bull. **48**, 506-642.

3. Bigelow HB, Schroeder WC (1953) Fishes of the Gulf of Maine.US Fish Wildlife Survey Fish. Bull. **53**, 1-577.

4. C. Rubio, Sobre el crecimiento, sexualidad y desarrollo gonadal de la mojarra rayada Eugerres plumieri (Cuvier), de la Cienaga Grande de Santa Marta con anotaciones sobre su biologõa. (Tesis Biol. Mar. Univ. Jorge Tadeo Lozano. Fac. Cienc. del Mar., Bogota, Columbia, 1975), p. 100.

5. Mook D, Copeia (1977) Larval and Osteological Development of Sheepshead, Archosargus probatocephalus (Pisces: Sparidae) **1**, 126-133.

6. Watson W (1983) Redescription of Larvae of the Pigfish, Orthopristis Chrysotera Linnaeus (Pisces, Haemulidae) Fish. Bull. **81**, 847-854.

7.Yáñez-Arancibia A, Lara-Domínguez AL (1988) Ecology of Three Sea Catfishes (Ariidae) in tropical coastal ecosystems – Southern Gulf of Mexico. Mar. Ecol. Prog. Ser. **49**, 215-230.

8. Warlen SM, J. S. Burke JS (1990) Immigration of Larvae of Fall/Winter Spawning Marine Fishes into a North Carolina Estuary. Estuaries **13**, 453-461.

9. Santos-Martinez A, Acero A (1991) Fish community of the Cienaga Grande de Santa Marta (Colombia): Composition and zoogeography. Ichthyol. Explor. Fres. **2**, 247-263.

10. Render JH, Wilson CA (1992) Reproduction Biology of Sheepshead in the Northern Gulf of Mexico. T. Am. Fish. Soc. **121**, 757-764.

11. Cervigón F (1994) Los peces marinos de Venezuela. Volume 3. (Fundación Científica Los Roques, Caracas,Venezuela), p. 295.

12. Collins LA, Johnson AG, Koenig CC, Baker MS, Jr. (1998) Reproductive Patterns, Sex Ration, and Fecundity in gag, Mycteroperca microlepis (Serranidae),a Protogynous Grouper from the Northeastern Gulf of Mexico. Fish. Bull. **96**, 415-427.

13. Hettler WF, Hare JA (1998) Abundance and Size of Larval Fishes outside the Entrance to Beaufort Inlet, North Carolina Estuaries **21**, 476-499 (1998).

14. Ruiz LJ, Figueroa MR, Prieto AA (1999) Ciclo Reproductive de Lactophrys quadricornis (Pisces: Ostraciidae) de la coasta nororiental de Venezuela. Rev. Biol. Trop. **47**, 561-570.

15. Holt GJ, Holt SA (2000) Vertical distribution and the role of physical processes in the feeding dynamics of two larval sciaenids Sciaenops ocellatus and Cynoscion nebulosus Mar. Ecol. Prog. Ser. **193**, 181-190.

16. Rogers JS, Hare JA, Lindquist DG (2001) Otolith Record of Age, Growth, and Ontogeny in Larval and Pelagic Juvenile Stephanolepis hispidus (Pisces: Monacanthidae). Mar. Biol. **138**, 945-953.

17. Nelson GA (2002) Age, growth, mortality, and distribution of pinfish (Lagodon rhomboides) in Tampa Bay and adjacent Gulf of Mexico waters. Fish.Bull. **100**, 582-592.

18. Wells RJD, Rooker JR (2004) Spatial and Temporal Patterna of Habitat use by Fishes Associated with Sargassum Mats in the Northwestern Gulf of Mexico. Bull. Mar. Sci. **74**, 81-99.

19. Fitzhugh GR, Koenig CC, Coleman FC, Grimes CB, Sturges W (2005) Spatial and temporal patterns in fertilization and settlement of young gag (Mycteroperca microlepis) along the West Florida Shelf Bull. Mar. Sci. **77**, 377-396.

20. Luczkovich JJ, Pullinger RC, Johnson SE, Sprague MW (2008) Identifying Sciaenid Critical Spawning Habitats by the Use of Passive Acoustics. T. Am. Fish. Soc. **137**, 576-605.

21. Malca E, Barimo JF, Serafy JE, Walsh PJ (2009)Age and growth of the gulf toadfish Opsanus beta based on otolith increment analysis. J. Fish. Biol. **75**, 1750-1761.

22. D’Alessandro EK, Sponaugle S, Serafy JE (2010) Larval ecology of a suite of snapper (family: Lutjanidae) in the Straits of Florida, western Atlantic Ocean. Mar. Ecol. Prog. Ser. **410**, 159-175.

23. Able KW, Fahay MP (2010) Ecology of Estuarine Fishes, Temperate Waters of the Western North Atlantic (The Johns Hopkins University Press), p. 566.
