# Supplementary material for: Response of Coastal Fishes to the Gulf of Mexico Oil Disaster
Source: PLoS One. 2011 Jul 6;6(7):e21609. doi: 10.1371/journal.pone.0021609 (PMC3130780; doi:10.1371/journal.pone.0021609)
Supplement: Table S5 — Summary table for the management status of the 20 most abundant fishes collected during our survey program. (DOCX) [file pone.0021609.s009.docx]

Table S5. Summary table for the management status of the 20 most abundant fishes collected during our survey program. Fishes that had a management plan in at least 1 state (LA, MS, AL, FL) or in Federal waters were listed as fished. These data, combined with available bycatch data from the northern Gulf of Mexico (references 1-8 below), were used to determine whether each species could have experienced a release from fishing pressure during the summer of 2010.

1. Watts NH, Pellegrin GJ (1982) Comparison of Shrimp and Finfish Catch

Rates and Ratios for Texas and Louisiana. Mar. Fish. Rev. **44**, 44-49.

2. Nichols S, Shah A, Pellegrin G, Mullin K (1987) Estimates of annual shrimp fleet bycatch for thirteen species in the offshore waters of the Gulf of Mexico. Report to the Gulf of Mexico Fishery Management Council, National Marine Fisheries Service, Southeast Fisheries Science Center, Pascagoula Laboratory, Pascagoula, MS, pp 27.

3. Nichols S, Shah A, Pellegrin G, Mullin K (1990) Updated estimates of shrimp fleet bycatch in the offshore waters of the Gulf of Mexico. Report to the Gulf of Mexico Fishery Management Council, National Marine Fisheries Service, Southeast Fisheries Science Center, Pascagoula Laboratory, Pascagoula, MS, pp 25.

4. Nichols S, Pellegrin G (1992) Revision and update of estimates of shrimp fleet bycatch 1972–1991. Report to the Gulf of Mexico Fishery Management Council, National Marine Fisheries Service, Southeast Fisheries Science Center, Pascagoula Laboratory, Pascagoula, MS.

5. Adkins GA (1993) Comprehensive assessment of bycatch in the Louisiana shrimp fishery. Louisiana Department of Wildlife and Fisheries, Marine Fisheries Division, Technical Bulletin No. 42.

6. Nichols S (2004) Some Bayesian approaches to estimation of shrimp fleet bycatch. Working paper to the Red Snapper Stock Assessment Data Workshop, April, 2004. Document No. SEDAR7-DW-3. Gulf of Mexico Fishery Management Council, Tampa, Florida.

7. Nichols S (2004) An update for the Bayesian estimation of shrimp fleet bycatch. Working paper to the Red Snapper Stock Assessment Data Workshop, April, 2004. Document No. SEDAR7-DW-54. Gulf of Mexico Fishery Management Council, Tampa, Florida.

8. Diamond SL (2004) Bycatch quotas in the Gulf of Mexico shrimp trawl fishery: can they work? Rev. Fish Biol. Fisher. **14**, 207-237.
