# Supplementary material for: Response of Coastal Fishes to the Gulf of Mexico Oil Disaster
Source: PLoS One. 2011 Jul 6;6(7):e21609. doi: 10.1371/journal.pone.0021609 (PMC3130780; doi:10.1371/journal.pone.0021609)
Supplement: Table S6 — Summary table of the effects of sampling area and year (context: pre- versus post-spill) on the catch rates of the 20 most abundant fishes collected during surveys in northern Gulf of Mexico seagrass meadows. (DOCX) [file pone.0021609.s010.docx]

Table S6. Summary table of the effects of sampling area and year (context: pre- versus post-spill) on the catch rates of the 20 most abundant fishes collected during surveys in northern Gulf of Mexico seagrass meadows. Significance results are based on 2-way ANOVAs.
