# Supplementary material for: Response of Coastal Fishes to the Gulf of Mexico Oil Disaster
Source: PLoS One. 2011 Jul 6;6(7):e21609. doi: 10.1371/journal.pone.0021609 (PMC3130780; doi:10.1371/journal.pone.0021609)
Supplement: Table S7 — Comparisons of community structure between catch data prior to (2006–2009) or immediately following (2010) the Deepwater Horizon disaster (ANOSIM and SIMPER). (DOCX) [file pone.0021609.s011.docx]

Table S7. Comparisons of community structure between catch data prior to (2006-2009) or immediately following (2010) the Deepwater Horizon disaster. Separate analyses were conducted for each survey region. Upper data show within-group similarity percentages calculated by SIMPER for 2006-2009 and 2010 data. Similarity percentages are followed by the three taxa most consistently collected (generally at high densities) in trawl samples within each region. Bottom table entries are pairwise dissimilarity percentages between groups (from SIMPER), including the five taxa most responsible for differences between groups. Chand Is, Global R = 0.052, Sig = 0.292; Gulf Is, Global R = 0.015, Sig = 0.367; Grand Bay, Global R = -0.123, 0.971; Florida Bays, Global R = 0.052, Sig = 0.072).
