# Supplementary material for: Response of Coastal Fishes to the Gulf of Mexico Oil Disaster
Source: PLoS One. 2011 Jul 6;6(7):e21609. doi: 10.1371/journal.pone.0021609 (PMC3130780; doi:10.1371/journal.pone.0021609)
Supplement: Table S8 — Summary table of the effects of sampling area and year (context: pre- versus post-spill) on the diversity (S, ES(20), H′, and J′) of trawl samples collected within northern Gulf of Mexico seagrass meadows. (DOCX) [file pone.0021609.s012.docx]

Table S8. Summary table of the effects of sampling area and year (context: pre- versus post-spill) on the diversity (S, ES_(20)_, H’, and J’) of trawl samples collected within northern Gulf of Mexico seagrass meadows. Significance results are based on 2-way ANOVAs.
