# Supplementary material for: Response of Coastal Fishes to the Gulf of Mexico Oil Disaster
Source: PLoS One. 2011 Jul 6;6(7):e21609. doi: 10.1371/journal.pone.0021609 (PMC3130780; doi:10.1371/journal.pone.0021609)
Supplement: Table S9 — Summary table of the effects of sampling area, larval risk and harvest pressure on the change in catch rates of individual species for pre- (2006–2009) and post-spill (2010) data. (DOCX) [file pone.0021609.s013.docx]

Table S9. Summary table of the effects of sampling area, larval risk and harvest pressure on the change in catch rates of individual species for pre- (2006-2009) and post-spill (2010) data (i.e., 2010 CPUE [Ave.] : 2006-2009 CPUE [Ave.]). Significance results are based on 2-way ANOVAs using sampling area and larval risk (top panel), or sampling area and harvest pressure (bottom panel) as fixed factors.
